# Angiogenic switch occurs late in squamous cell carcinomas of human skin

**DOI:** 10.1054/bjoc.1999.0969

**Published:** 2000-01-18

**Authors:** S Strieth, W Hartschuh, L Pilz, N E Fusenig

**Affiliations:** Division of Differentiation and Carcinogenesis and Central Unit of Biostatistics, German Cancer Research Center (DKFZ), Im Neuenheimer Feld 280, Heidelberg, 69120, Germany; Department of Dermatology, University Hospital, Heidelberg, Germany

**Keywords:** angiogenesis, human skin SCC, vascularization, tumour stages, VEGF

## Abstract

Angiogenesis is a crucial event in carcinogenesis and its onset has been associated with premalignant tumour stages. In order to elucidate the significance of angiogenesis in different stages of epithelial skin tumours, we analysed the vessel density in ten normal skin samples, 14 actinic keratosis (AK), 12 hypertrophic AKs, and in nine early- and 16 late-stage squamous cell carcinomas (SCCs). Mean vascular density was quantitated by counting the number of CD 31-immunostained blood vessels and by morphometric assessment of stained vessel area by computer-assisted image analysis. The results from both methods were well correlated. Mean vascular density was similar in normal dermis and in AK, and only slightly elevated in hypertrophic AKs and early SCC stages (tumour thickness < 2 mm). Only late-stage SCCs infiltrating the subcutis exhibited a significant increase in vascularization. Vessel density was independent of tumour localization, degree of proliferation and inflammatory cell infiltration. Furthermore, tumour vascularization was not correlated with the expression of vascular endothelial growth factor, a major angiogenic factor, as revealed by in situ hybridization and immunohistochemistry. The restriction of enhanced vascularization to increased tumour thickness may be a major reason for the rather low metastatic spread of cutaneous SCCs. © 2000 CancerResearch Campaign


British Journal of Cancer (2000) 82(3), 591–600
© 2000 Cancer Research Campaign
DOI: 10.1054/ bjoc.1999.0969, available online at http://www.idealibrary.com on IDEAL®

### Angiogenic switch occurs late in squamous cell carcinomas of human skin

**S Strieth[1], W Hartschuh[2], L Pilz[3] and NE Fusenig[1]**

[1]Division of Differentiation and Carcinogenesis and [3]Central Unit of Biostatistics, German Cancer Research Center (DKFZ), Im Neuenheimer Feld 280, 69120 Heidelberg, Germany; [2]Department of Dermatology, University Hospital, Heidelberg, Germany

**Summary** Angiogenesis is a crucial event in carcinogenesis and its onset has been associated with premalignant tumour stages. In order to elucidate the significance of angiogenesis in different stages of epithelial skin tumours, we analysed the vessel density in ten normal skin samples, 14 actinic keratosis (AK), 12 hypertrophic AKs, and in nine early- and 16 late-stage squamous cell carcinomas (SCCs). Mean vascular density was quantitated by counting the number of CD 31-immunostained blood vessels and by morphometric assessment of stained vessel area by computer-assisted image analysis. The results from both methods were well correlated. Mean vascular density was similar in normal dermis and in AK, and only slightly elevated in hypertrophic AKs and early SCC stages (tumour thickness < 2 mm). Only late-stage SCCs infiltrating the subcutis exhibited a significant increase in vascularization. Vessel density was independent of tumour localization, degree of proliferation and inflammatory cell infiltration. Furthermore, tumour vascularization was not correlated with the expression of vascular endothelial growth factor, a major angiogenic factor, as revealed by in situ hybridization and immunohistochemistry. The restriction of enhanced vascularization to increased tumour thickness may be a major reason for the rather low metastatic spread of cutaneous SCCs.
© 2000 Cancer Research Campaign

**Keywords:** angiogenesis; human skin SCC; vascularization; tumour stages; VEGF

It is now well established that malignant tumours critically depend on neovascularization for continued growth, invasion and formation of metastases (Folkman, 1995). Obviously, new vascularization promotes tumour growth by improving the exchange of nutrients, oxygen and waste products. There is increasing evidence that, in addition to this perfusion effect, endothelial cells release important paracrine growth factors for tumour cells (Hamada et al, 1992; Weidner, 1995; Rak et al, 1996). Also, the invasive behaviour of endothelial cells at the tips of growing capillaries is facilitated by their secretion of matrix-degrading enzymes, which are likely to facilitate spreading of tumour cells into the connective tissue (Pyke et al, 1991). Thus, by the additive impact of the perfusion and paracrine growth effects with the production of proteases, activated endothelia may significantly contribute to tumour growth, invasion and metastasis.

Within the last decade a number of studies on tumours of the breast, cervix, prostate and of malignant melanoma (reviewed in Weidner, 1995) have revealed a correlation of increased intratumour microvessel density with different parameters of tumour aggressiveness such as relapses, formation of metastases, or decreased patient survival. However, there have also been contradictory results showing no such correlations (Carrau et al, 1995; Tahan and Stein, 1995) and it is not clear whether this discrepancy is due to intratumoural heterogeneity, tumour type and localization or differences in analytical methods. Notably, many of these studies which did not demonstrate a correlation between tumour vessel density and progression had analysed squamous cell carcinomas (SCCs) of the head and neck (Carrau et al, 1995; Tahan and Stein, 1995).

*Received 8 March 1999*
*Revised 12 July 1999*
*Accepted 15 July 1999*

*Correspondence to:* NE Fusenig

In skin, the prognostic significance of tumour vascularity for a higher probability to progress to more advanced tumour stages has been documented for melanomas, in particular for those exhibiting intermediate thickness and being classified as ‘low risk tumours’ (Srivastava et al, 1988; Marcoval et al, 1997). As far as SCCs are concerned, there are small amounts of data available of tumours of the skin (Urbach and Graham, 1962; Tahan and Stein, 1995; Weninger et al, 1996, 1997) which do not show a strong correlation of angiogenesis with tumour progression except for basal cell carcinomas (Staibano et al, 1996).

Although it is common finding that malignant tumours have increased vascularization, less is known about the regulation of the ‘switch to the angiogenic phenotype’ (Hanahan and Folkman, 1996). In particular, it is not clear whether this crucial step in tumour development generally precedes the conversion of premalignant to malignant neoplasms, as data from transgenic mouse models have shown (Coussens et al, 1996; Smith-McCune et al, 1997). Patient data from mammary gland, uterine cervix and prostate have confirmed these experimental data, demonstrating the onset of increased vascularization in premalignant stages, mostly in intraepithelial neoplasia (Brawer et al, 1994; Guidi et al, 1994; Smith-McCune and Weidner, 1994).

In skin, as in other organs, epithelial neoplasia develops through preneoplastic stages, but little is known about the association of angiogenesis with these different stages of epithelial carcinogenesis. In humans, 60% of SCCs of the skin develop through defined stages called actinic or solar keratosis (AK). These premalignant lesions exhibit up to 20% probability of becoming malignant (Marks et al, 1988; Callen, 1991) and carry similar genetic changes as found in SCCs, such as mutations in the p53 gene (Ziegler et al, 1994). The AK can be graded into 'basic' and more progressive, hypertrophic or proliferating subtypes (Weedon, 1997), the latter being also classified as carcinoma in situ (Callen, 1991). Little is known about the cellular and molecular changes associated with the transition of premalignant to malignant (invasive) skin lesions in humans. Experimental in vitro models have been developed and different stages in the carcinogenesis process characterized at the genetic and phenotypic level (Boukamp et al, 1995; Fusenig and Boukamp, 1998). Distinct patterns of angiogenesis in heterotransplants in nude mice (Skobe et al, 1997) indicated that persistent angiogenesis and increased vascularization are restricted to malignant tumours in this model. However, comparable in situ studies on SCCs of human skin are lacking correlate to the onset of tumour angiogenesis with distinct stages in skin cancer development (Urbach and Graham, 1962).

In order to analyse the angiogenic switch in epithelial skin tumour development, we examined vascular density in the stroma of normal skin, AK, its hypertrophic form, and early- as well as late-stage SCCs. As the angiogenic switch is considered a result of an imbalance between angiogenesis inductors and inhibitors (Hanahan and Folkman, 1996), we further investigated the expression of the most potent angiogenic factor vascular endothelial growth factor (VEGF) (Folkman, 1995; Weninger et al, 1996) at the RNA and protein level. We here demonstrate that in epithelial skin carcinogenesis tumour vascularization is significantly increased only in later stage SCCs and that this is not correlated with a different expression level of VEGF in these lesions.

### MATERIALS AND METHODS

### Patients and materials

Formalin-fixed, paraffin-embedded specimens from 51 patients with different epithelial lesions were analysed including AK (14), its hypertrophic form (12) and SCCs (25) obtained for therapeutic purposes with fully informed consent of patients (Table 1). The SCCs were subdivided into two groups following the classification of Breuninger (1990) on the basis of tumour thickness and depth of infiltration. According to this microstaging of SCCs of the skin, nine SCCs had a tumour thickness of less than 2 mm and were still confined to the corium and thus classified as early stages. Sixteen cases had a thickness of more than 2 mm and their invasive front extended into the subcutaneous fat, and were thus classified as late-stage SCCs exhibiting a higher probability to metastasize (Breuninger, 1990). Two SCCs of the lip were also included in this group of late-stage SCCs although their thickness was less than 2 mm, but they had infiltrated the subcutaneous fat. In addition, five recurrent tumour specimens were included: two of each belonging to the groups of early and late SCCs and one was diagnosed as hypertrophic AK. Significant neurotropism (perineural spread) often associated with high recurrence and metastasis rate (Goepfert et al, 1984) was not observed in any of the tumours. These tumour specimens were compared with ten normal skin biopsies of comparable anatomical locations obtained for therapeutic purposes (skin transplantation, tumour periphery) exhibiting no histopathological alterations. Haematoxylin and eosin (H&E)-stained sections of the paraffin-embedded blocks were reviewed and the tumour stages classified by a dermatopathologist not involved in the analysis of angiogenesis. In none of the patients had metastases been observed for a period of more than 5 years.

**Table 1** Classification of stages of skin carcinomas

| | *n* | Depth of infiltration | Tumour thickness | Broders' grading |
|---|---|---|---|---|
| Normal skin | 10 | – | | |
| Actinic keratosis | 14 | – | | |
| Hypertrophic a. k. (carcinoma in situ) | 12 | – | | |
| Early SCC | 9 | Corium | <2 mm | I |
| Late SCC | 16 | Subcutaneous fat | >2 mm | I–IV |

### CD31 immunohistochemistry

Sections (5-mm thick) were cut from the retrieved tumour blocks, dewaxed and rehydrated in graded ethanol. Endogenous peroxidase was blocked with 0.3% hydrogen peroxide for 15 min. To unmask hidden epitopes, sections were digested with protease type XXIV (Sigma, Deisenhofen, Germany; 10 mg $ml^{-1}$) at 37°C for 15 min. Vessels were visualized by immunostaining with a monoclonal antibody against CD31 (clone JC70/A; Dako, Glostrup, Denmark). Sections were incubated for 6 h (the optimal time) with the primary antibody diluted 1:40 in phosphate-buffered saline (PBS) and subsequently developed using a streptavidin–biotin–peroxidase system (Amersham, Braunschweig, Germany). Visualization of the antibody complex was achieved with a nickel-enhanced diaminobencidin reaction (Hsu et al, 1982) resulting in black staining of endothelial cell membranes. Sections were counterstained by a modified Masson–Goldner protocol omitting haematoxylin.

### Quantitation of vessel density

Sections were screened at a magnification of 100× for areas of highest microvessel density, so-called 'hot spots' (Weidner et al, 1991; Van Hoef et al, 1993) and three such areas were analysed per section. While focusing on the margins of epithelial tissues, areas next to hair follicles and glands were avoided. At a magnification of 200× the black-stained vessels were counted in a square grid defining an area of 0.09 $mm^2$ (349 μm × 264 μm). All black-stained single cells or clusters of cells which were separated from other stained areas and vessels of all sizes were counted. Lymphatic vessels were not marked by the antibody. Vascular density was defined as the mean vessel count of three areas analysed per section. Results obtained from different sections of the same tumour showed good reproducibility (data not shown). Additionally, vessel density was determined by computer-assisted image analysis in the same grid-defined area used for the manual count at a magnification of 200× using a Leica Quantimet 600QWin-Image Analysis System equipped with a 3CCD camera. The black-stained vessels were detected automatically using the Hue-Saturation-Intensity (HSI) system. In cases where interference was observed with other dark material such as erythrocytes or horn material, this was corrected interactively. Vascular density was calculated as the mean of three measurements and blotted as area per cent.

 © 2000 Cancer Research Campaign

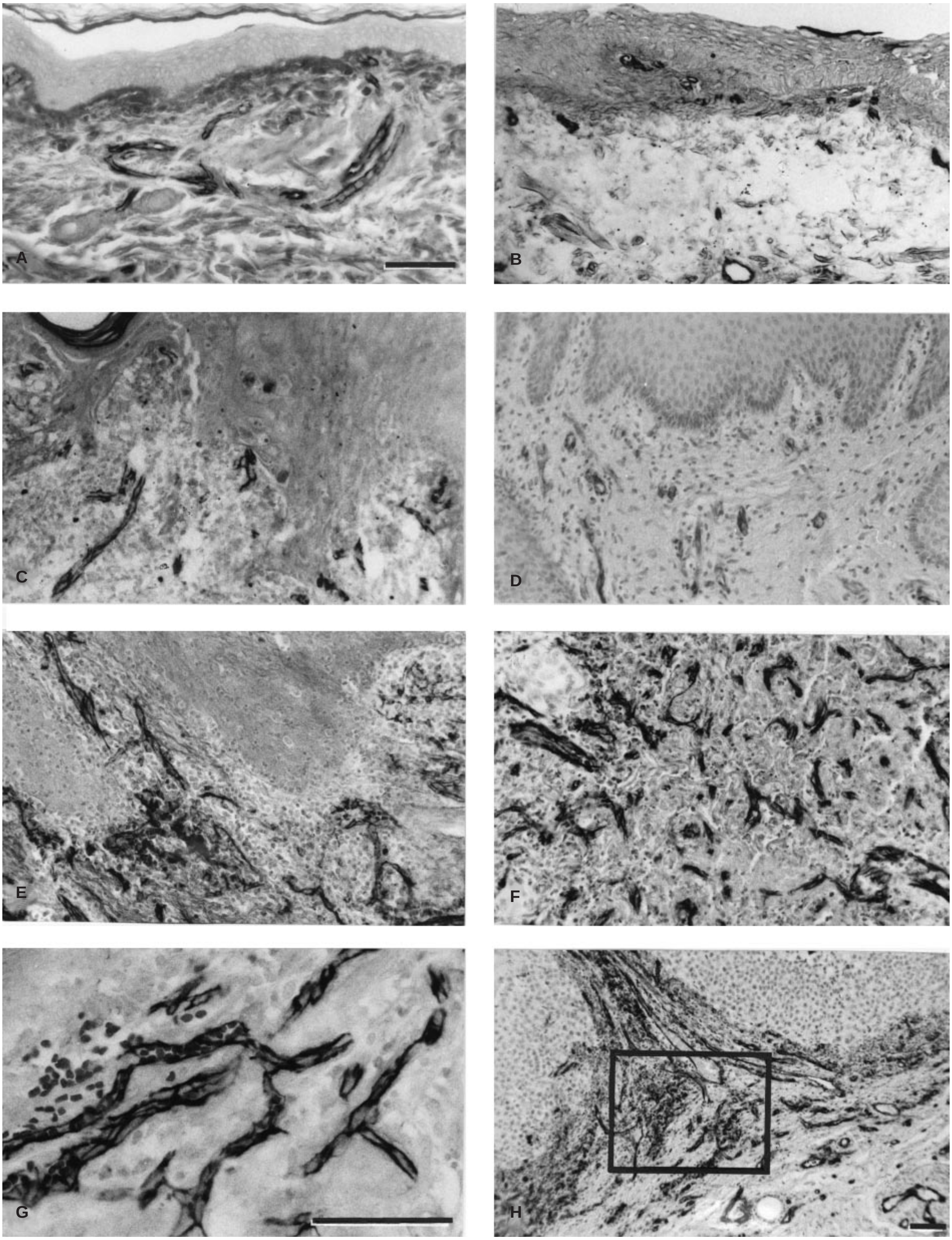

**Figure 1** Immunohistochemical staining of capillaries by a CD 31 antibody in: the dermis of normal skin (**A**), actinic keratosis (AK) (**B**) (most of the keratotic material was lost during processing), in the hypertrophic form of AK (**C**), in early-stage squamous cell carcinoma (SCC) (**D**), and a well differentiated (**E**) as well as undifferentiated (**F**) late-stage SCC. Small and large blood vessels are clearly discernible by staining of endothelial cell membranes (**G**). Vascular density was assayed in 'hot spot' areas at the epithelial–stromal border in a frame of 349 μm × 264 μm as shown in an SCC section (**H**) at lower magnification. Magnification in **A–F** 144×, in **G** 288×, in **H** 72× (bars: 23 μm). Counterstaining in **A, B, C, E, G** with a modified Masson–Goldner protocol; in **D, F, H** with haematoxylin

*© 2000 Cancer Research Campaign* 

### Statistical analysis

The mean area percentage and the mean manual count data have been analysed with the MS Excel software and the statistics program ADAM. The Wilcoxon rank sum test with multiple comparison was used to test differences in vascular density values (vessel count/vessel area) of the five tumour stages at a significance level of 0.01. The high level of significance was chosen to compensate for the multiple comparison of five groups (adjusting the $\alpha$-parameter).

### Assessment of chronic inflammatory cell infiltrate

Parallel H&E-stained sections were assessed for inflammatory infiltration consisting mainly of lymphocytes. Cell density was screened by a pathologist not involved in determining vessel density and classified as no infiltration (–), low degree (+), intermediate degree (++) and strong degree of infiltration (+++).

### Determination of proliferative activity

Parallel sections of normal skin and four early as well as seven late SCCs were analysed for proliferating cells by immunostaining with the monoclonal antibody Mib-1 (Ki-67; Dianova, Hamburg, Germany) after 30 min microwave incubation and using the same immunohistochemical method (DAB-nickel) as for the identification of endothelial cells. At least 100 proliferating cells per specimen were counted in basal layers and zones of proliferation respectively.

### In situ hybridization of VEGF-RNA

Human cDNA (649 bp) encoding human $VEGF_{165}$ in Bluescript $KS^-$ vector (Stratagene) (Keck et al, 1989), generously provided by Drs H Weich and D Marmé (Freiburg, Germany) was used as a probe. The in situ hybridization was essentially performed as described (Moorman et al, 1992). In brief, $^{35}S$-labelled RNA probes for VEGF were prepared using T3 or T7 RNA-polymerase (for antisense and sense respectively) according to the manufacturer's instructions (Boehringer Mannheim). Three paraffin sections per tumour stage, parallel to those used for determining vascular density, were pretreated, hybridized and washed at high stringency as described (Moorman et al, 1992). For autoradiography, slides were coated with NTB-2 film emulsion and exposed for 4 weeks. After the film was developed, the sections were counterstained with haematoxylin.

### VEGF immunohistochemistry

Indirect immunohistochemistry using a polyclonal antibody against $VEGF_{165}$ (1:20; Calbiochem, Cambridge, Cat. No. GF 25) was performed on parallel sections to those specimens used for in situ hybridization. Pretreatment of sections was performed by a 30 min incubation in 0.05% Saponin (Sigma, Deisenhofen, Germany). Background staining was blocked by incubating the slides 15 min in streptavidin (1 $\mu$g $ml^{-1}$; Sigma, Deisenhofen, Germany) and normal goat serum (1:10, Dianova, Hamburg, Germany) before application of the primary antibody. The secondary antibody was diluted in PBS with 5% human serum (Dianova, Hamburg, Germany). Recombinant $VEGF_{165}$ (*Escherichia coli*), kindly provided by Dr D Marmé (Freiburg, Germany), was used for neutralizing the antibody as a negative control (ligand/antibody = 9.7/1, based on protein mass). Evaluation of RNA and protein expression of VEGF was based both on the area and intensity of staining.

### RESULTS

### Selected tumour stages of skin SCCs

Different stages of premalignant lesions and SCCs were classified on the basis of H&E-stained sections by a dermatopathologist (Table 1) and characteristic tumour stages selected for analysis of vessel density. In addition to normal unaffected skin (Figure 1A), actinic keratoses were analysed. They were subdivided into two classes based on their degree of hyperplasia and dysplasia. The first representing the typical appearance of a hyperparakeratotic epithelium of more or less atypical keratinocytes lacking the normal stratified arrangement of the epidermis (Figure 1B). Following the definitions of Weedon (1997) the hypertrophic form of AK (also called proliferating AK) was characterized by a hyperplastic epithelium exhibiting a higher degree of dysplasia and deeper indentations of atypical basal keratinocytes into the dermis (Figure 1C). Invasive SCCs were subdivided in low-risk and higher risk SCCs depending on their tumour thickness and depths of infiltration (Breuninger et al, 1990) and classified as early stages (< 2 mm thickness) (Figure 1D) and late-stage SCCs (> 2 mm) (Figure 1 E,F). The latter lesions usually have a moderate tendency for metastasis, whereas metastatic probability increases strongly when skin SCCs reach a thickness of more than 6 mm (Breuninger et al, 1990). Most SCCs had a moderate to low degree of differentiation with only a few being of an undifferentiated phenotype (Figure 1F). However, no distinction was made between these subtypes according to Broders' (1932) classification.

### Immunohistochemical detection of blood vessels

When comparing different staining procedures for identifying blood vessels using antibodies against factor VIII-related Ag, collagen type IV and CD31/PECAM respectively, the latter gave the best and most consistent results. With CD31 antibodies staining was most uniform, intense, and with no or very low background (see Figure 1). Using the nickel enhancement procedure endothelial cells forming the walls of smaller and larger vessels could be easily identified and even very small vessels clearly recognized (Figure 1G). Vessels were counted and the density of the stained area morphometrically assessed in three framed areas per section choosing 'hot spots' of vascular density as indicated in Figure 1H.

### Late angiogenic switch in skin carcinogenesis

In sections of anatomically related normal skin, a typical capillary bed of a normal, uninflamed and non-irritated dermis was visible (Figure 1A). Increased vascular density was usually noticed around hair follicles and sebaceous glands, but these areas were excluded. In AK, both in the 'classical' and the hypertrophic form, the thickened epidermis was in some cases lined with increased vessel density but this was confined to a small subepidermal zone

© 2000 Cancer Research Campaign

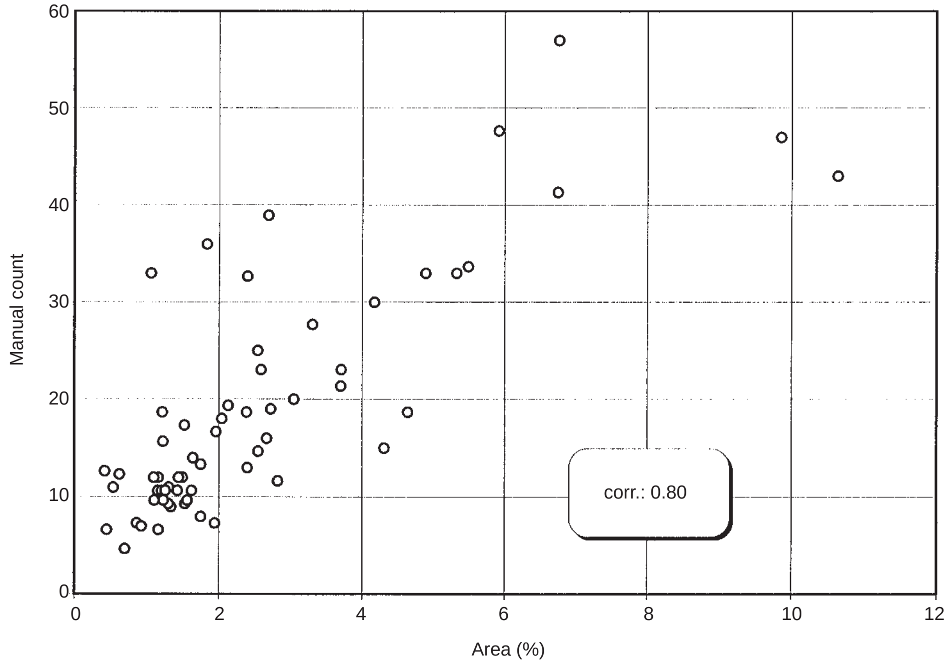

**Figure 2** Correlation of manual counts and computer-assisted quantitation of stained area of all specimens yielding a correlation coefficient of 0.80

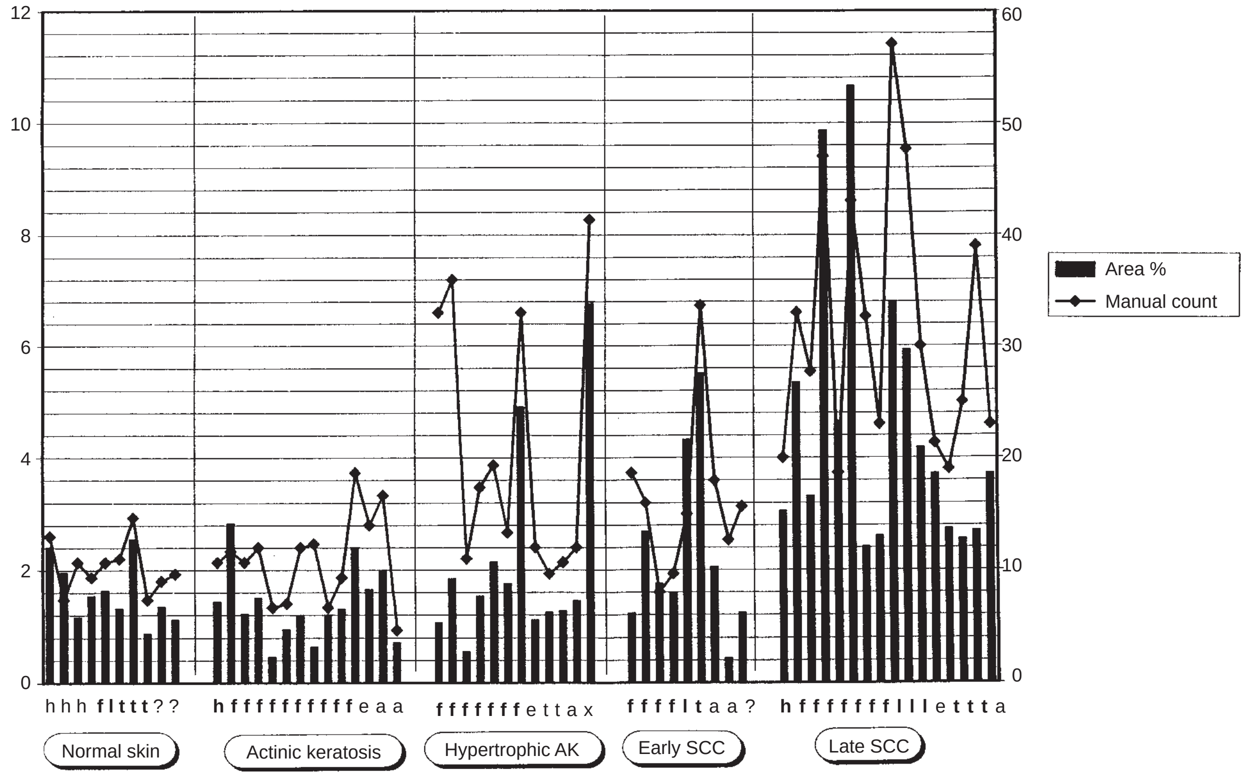

**Figure 3** Vascular density in different skin lesions analysed by computerized measurements of stained areas (■) and manual count (–◆–). The localization of the different lesions in h: head, f: face, l: lip, e: ear, t: trunk, a: arm, x: leg, ?: not reported

© 2000 Cancer Research Campaign

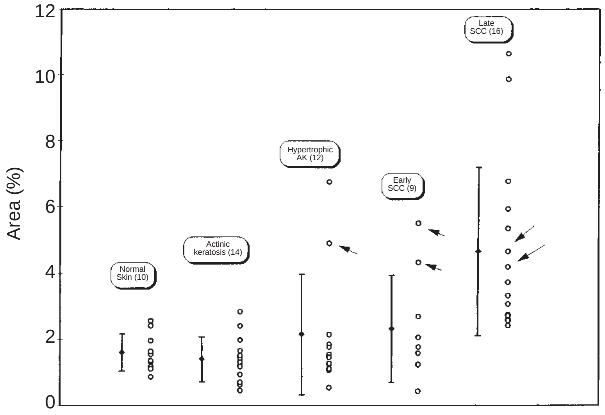

**Figure 4** Statistical evaluation of vascular density as measured by computerized stained area analysis in the different skin lesions. Both single values (○) as well as calculated means (◆) with standard deviation are given. Three of the four strongly vascularized lesions of hypertrophic AKs and early SCCs as well as two late SCCs were identified as recurrent lesions (arrows)

(Figure 1 B,C). In early-stage SCCs the invading tumour tips were surrounded by a net of small- and medium-sized vessels which sometimes covered a larger area, while generally resembling those seen in AK (Figure 1D). Vascularization was clearly increased in late-stage SCCs and particularly obvious in so-called 'hot spots', irrespective of whether tumours were keratinized or undifferentiated (Figure 1 E,F).

Both methods of quantitation: manual count and computer-assisted analysis of stained area showed a good correlation coefficient of 0.80 (Figure 2) instead of some variations in vessel staining due to different vessel size and section planes (see Figure 1). The close correlation was similarly obvious when the manual counts were blotted together with the area measurements for the different tumour stages (Figure 3). With few exceptions, the values of both evaluation methods were very similar, although some individual variations were visible in the three tumour groups. Nevertheless, for every group high correlations were calculated (normal skin: 0.68; AK: 0.64; hypertrophic AK: 0.98; early SCC: 0.89; late SCC: 0.81).

Quite remarkably, the assessed vessel density did not differ much between normal skin and early SCCs irrespective of localization (Figures 3 and 4). Three of the four specimens with higher vascularizations in hypertrophic AK and early SCC (arrows) were identified as locally recurrent tumours known to behave more aggressively than the primary lesion. In late-stage SCCs, however, vascular density was clearly increased above the level of the premalignant stages. When calculated by the Wilcoxon rank sum test with multiple comparison, a significant difference in vascular density was evident only in late-stage SCCs (late SCC vs normal skin and AK: $P < 0.00005$; vs hypertrophic AK: $P = 0.0004$, and vs early SCC: $P = 0.0002$). No other group of lesions exhibited significant differences in vessel density when compared with normal skin or between the different groups (AK vs normal skin: $P = 0.4366$; hypertrophic AK vs normal skin: $P = 1.000$; vs AK: $P = 0.3217$; early SCC vs normal skin: $P = 0.3562$; vs AK: $P = 0.1245$; vs hypertrophic AK: $P = 0.6511$).

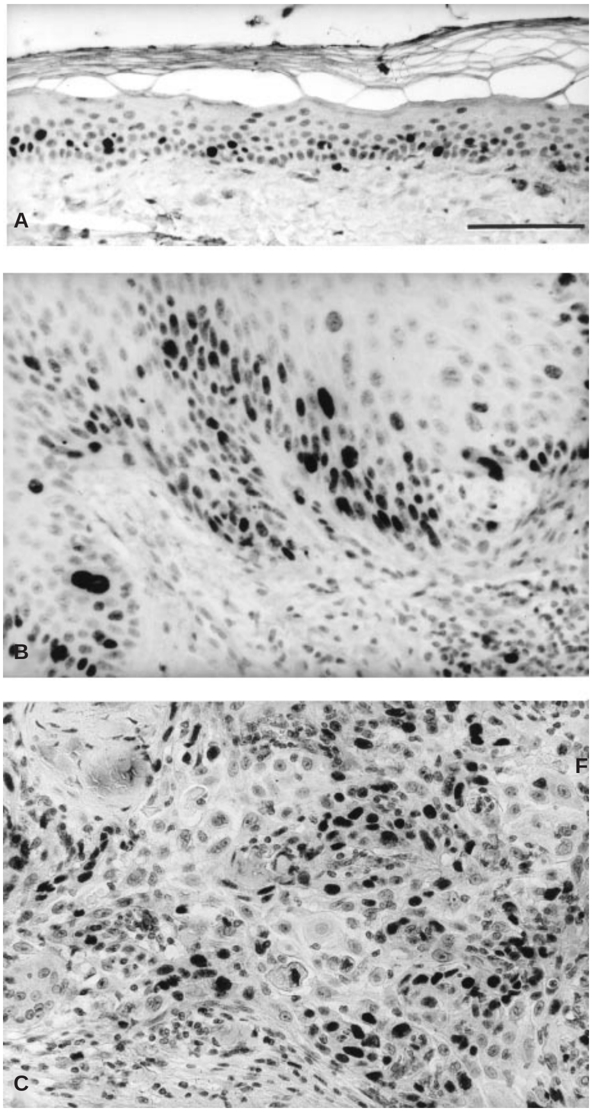

**Figure 5** Proliferating cells identified as labelled nuclei stained by the Mib-1 (Ki67) antibody in normal epidermis (**A**), early (**B**) and late (**C**) SCCs (haematoxylin, magnification **A–C** 288×; bar: 23 µm)

### Impact of chronic inflammatory cell infiltrates and tumour cell proliferation

In order to exclude that changes not associated with tumour stage would have contributed to the observed vascular density in the tumour stroma, the frequency of white blood cells, mainly lymphocytes, was assessed by an arbitrary scale on H&E-stained sections at a magnification of 200×. While there was essentially no inflammatory cell infiltrate in normal skin, this level was clearly higher, though of varying intensity, in actinic keratosis and in early- as well as late-stage SCCs. However, there was no correlation between the levels of inflammatory cell infiltration and vascular density as determined on parallel sections both when overall and focal intensities were compared. This documented that vessel density was not significantly affected by inflammatory cell

© 2000 Cancer Research Campaign infiltrates. In addition, no basic disease of the tumour-bearers (e.g. diabetes or psoriasis) was evident which could have effected vascular density.

When the rate of proliferating (Mib-1-positive) cells was assessed in selected specimens of early and late SCCs (Figure 5), there was a visible increase in proliferating cells in late SCCs (early SCC: 38% ± 5%; late SCC 52% ± 11%), which, however, was statistically not significant. Kerschmann et al (1994) also reported a positive trend but no significant change in mean proliferation fractions according to increasing tumour grades. This indicated that the significant higher rate of vascularization in these more advanced tumour stages was not associated with a higher proliferative activity of tumour cells. In particular, individual tumours of late SCCs with highest vascular density did not exhibit the highest proliferative activity so that both parameters were not correlated.

### Expression of VEGF RNA and protein

In order to determine whether the increased vascular density of late stage SCCs was caused by an elevated synthesis of an angiogenic inducer, the RNA and protein levels of the most potent angiogenic factor, the VEGF were analysed in parallel sections. By radioactive in situ hybridization strong VEGF expression in epithelial cells could be detected, however, no differences were obvious between premalignant and malignant lesions (Figure 6). Expression was similarly intense in AK (Figure 6A), its hypertrophic form (not shown) as well as early- (Figure 6B) and late-stage SCCs (Figure 6C), while the sense probe only yielded some background labelling (Figure 6D). The epidermis of normal skin also expressed VEGF-RNA, although at a lower level than the tumour lesions.

Label was observed in all epidermal strata, though with a tendency to more intensity in differentiated layers, and in SCCs it was rather homogeneously distributed with no distinct inter-tumoural variability.

Comparably, VEGF protein was detected by indirect immunohistochemistry in similar intensity in AK (not shown), its hypertrophic form (Figure 6E) and in early (Figure 6F) and late SCCs (Figure 6G). In normal skin the staining of the protein was very weak.

Again the staining was more intense over differentiated cells and there was a homogenous staining pattern in each group. As control for the antibody specificity, the immunoreaction was abolished, as shown for late-stage SCCs by blocking the antibody with recombinant human VEGF (Figure 6H).

### DISCUSSION

A considerable body of research has documented that tumour growth and metastasis require persistent new blood vessel growth (Folkman, 1995). Based on the frequently observed correlation between angiogenesis and rapid tumour expansion the question has been raised as to when angiogenesis is activated in the course of tumour development and whether the onset of tumour angiogenesis can be a diagnostic parameter (Srivastava et al, 1988; Carrau et al, 1995; Weidner, 1995; Staibano et al, 1996). There is increasing evidence that angiogenesis is induced during the early, preneoplastic stages of tumour development (Hanahan and Folkman, 1996). The clues initially came from experimental carcinogenesis models using transgenic mice which express distinct stages of tumour development (Coussens et al, 1996; Smith-McCune et al, 1997). In three different mouse models of carcinogenesis including islet cell carcinoma, dermal fibrosarcoma and epidermal SCC, the angiogenic switch occurred during early stages preceding the appearance of the solid tumours (Hanahan and Folkman, 1996). This suggested that the regulation of angiogenesis is an important, potentially rate-limiting step in the pathway to many solid tumours.

In human cancer, vessel density in invasive tumours has been demonstrated to be a significant prognostic indicator for both breast (Weidner et al, 1991) and cervical cancers (Schlenger et al, 1995). In both mammary duct and uterine cervix the angiogenic switch occurred before the tumour started to invade (Guidi et al, 1994; Smith-McCune and Weidner, 1994). The level of angiogenesis in the premalignant lesions did not correlate with the degree of inflammation but with the grade of intraepithelial neoplasia. From these as well as the experimental data it was suggested that the ability to induce angiogenesis precedes the conversion of premalignant to malignant tumours (Hanahan and Folkman, 1996).

In contrast to these observations, our findings demonstrate that in skin epithelial neoplasia a significant increase in vascularization occurs only in later stage SCCs. Even more astonishingly, the mean vessel density in the premalignant lesions of actinic keratosis and early stages of invasive SCCs does not differ significantly from that in the dermis of normal skin. The few exceptions with high vascular density among the early skin lesions are found in recurrent tumours and may either be due to previous treatment or increased malignancy of the recurrent tumour. These findings indicate that in epithelial skin malignancies the angiogenic switch occurs late during tumour progression and not in premalignant and pre-invasive stages.

This delayed onset of increasing tumour vascularization was not correlated to the level of expression of the most potent angiogenic factor VEGF. The expression levels of VEGF (both at the RNA and protein level) were similar in all lesions from the premalignant actinic keratoses to the late-stage carcinomas. This is in agreement with earlier reports, demonstrating VEGF expression in malignant skin carcinoma as well as benign tumours, such as keratoakathoma and even in normal epidermis (Detmar et al, 1994; Weninger et al, 1996). Moreover, VEGF expression was observed in epidermal hyperplastic diseases, such as psoriasis and is supposedly induced by hypoxic conditions in the expanded epithelia (Detmar et al, 1997). The functional significance of VEGF in angiogenesis associated with healing skin wounds (Brown et al, 1992) as well as its function on dermal vascular endothelial cells (Detmar et al, 1995) has been documented. In contrast to Weninger et al (1996) who described a membrane staining of keratinocytes we found a cytoplasmatic staining pattern for the VEGF protein especially in suprabasal cell layers, a pattern comparable to that described by Detmar et al (1994, and references therein) using the same antibody. The lack of correlation between VEGF expression and tumour stages might indicate that this angiogenic factor may not be the rate-limiting step for the increased tumour vascularization. This may be determined by other factors as well and depend on the expression of their respective receptors on vascular endothelial cells. Recent studies on an experimental skin carcinogenesis model had demonstrated a decisive role of VEGF receptor 2 expression for malignancy-associated angiogenesis (Skobe et al,

© 2000 Cancer Research Campaign 

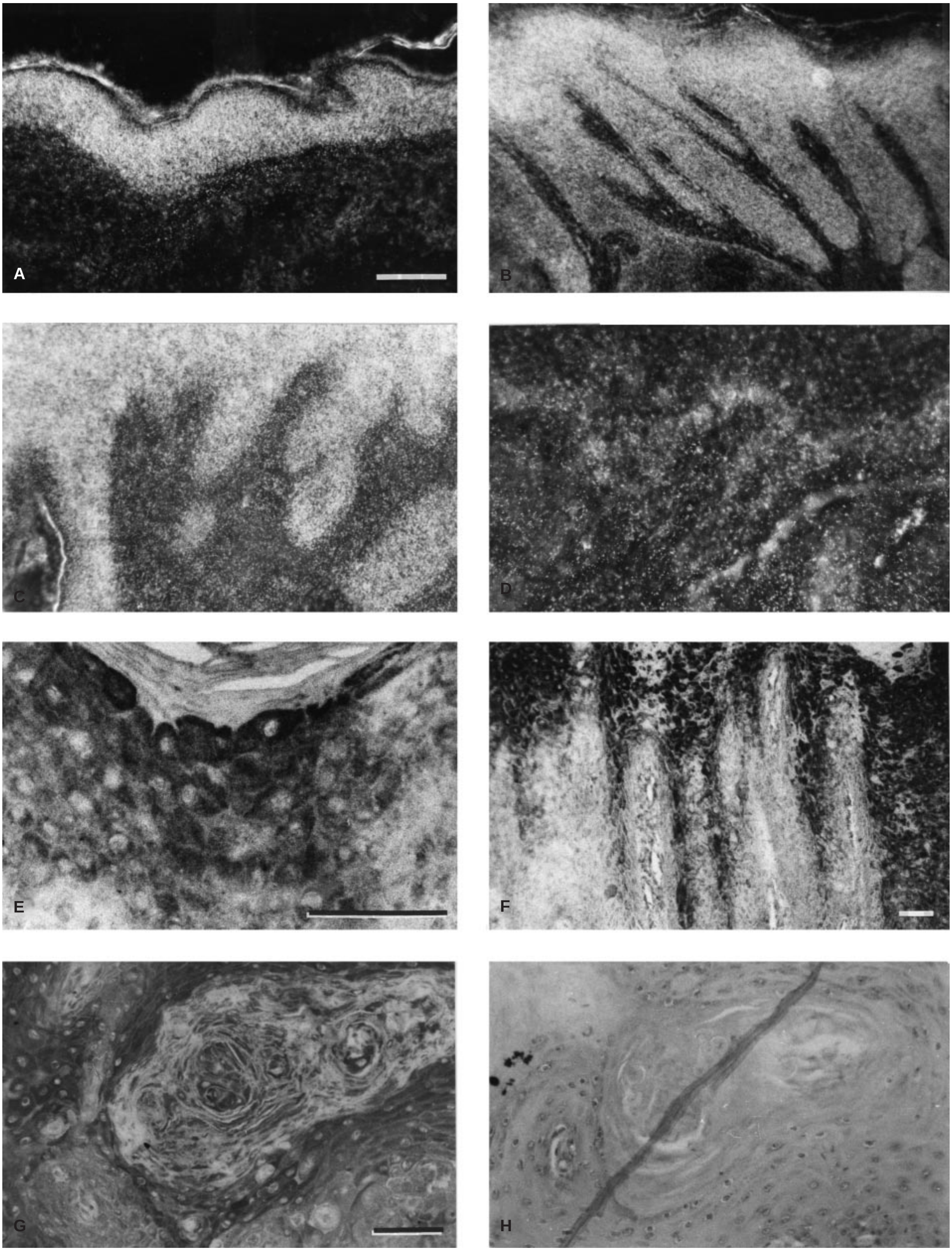

**Figure 6** Expression of VEGF as revealed by radioactive in situ hybridization in actinic keratosis (**A**), early SCC (**B**) and late SCC (**C**); **D**: corresponding sense probe control. Protein localization by immunohistochemistry revealed a similar distribution with preference to differentiating cells in hypertrophic AK, **E**; early SCC, **F**; late SCC, **G**; as a control, in a late SCC (**H**) staining was abolished after absorption of the antibody with recombinant VEGF (haematoxylin-counterstaining, magnification **A–D, G–H**: 144×, **E**: 288×, **F**: 72×; bars: 23 µm)

*© 2000 Cancer Research Campaign*

1997). While VEGFR-2 was only transiently activated in transplants of benign keratinocytes, it was continuously up-regulated in the endothelial cells of malignant stroma concomitant with ongoing angiogenesis and invasion. Moreover, the functional significance of this correlation was proven by blocking VEGF/VEGFR-2 interaction resulting in drastic reduction of vascularization accompanied by abrogation of tumour cell invasion (Skobe et al, 1997). The mechanism of VEGFR-2 activation and whether this process plays a decisive role in the late onset of skin SCC angiogenesis remains to be determined.

So far, there have been no reports studying the onset of angiogenesis in epithelial skin tumour development in man. A higher vascular density has been observed in more aggressive than in relatively benign basal cell carcinomas (Staibano et al, 1996). Squamous cell carcinomas showed a higher microvessel density than basal cell carcinomas (Weninger et al, 1996), but similar vascularization than the benign keratoakanthomas (Weninger et al, 1997). However, in these studies no comparison with tumour stages of SCCs and vascular density has been made.

This is in contrast to malignant melanoma, where increased angiogenesis was observed with the transition from the radial-growth-phase tumours (usually having a good prognosis) to the vertical-growth-phase (Marcoval et al, 1997). Thus, a high vascular count in thin primary melanomas is considered predictive of fatal metastatic disease (Barnhill et al, 1992; Barnhill and Levy, 1993; Graham et al, 1994). Similarly, in SCCs of the head and neck, correlations between microvessel density, bad prognosis and metastatic probability (Gasparini et al, 1993; Zätterström et al, 1995) have been reported, while others found no correlation between angiogenesis and tumour stage or prognosis (Carrau et al, 1995; Tahan and Stein, 1995). Whether these discrepancies are due to the analysis of different tumour stages, localizations, or methods of analysis, is not clear.

There has been a long debate on the best way to assess tumour vascularization (Vermeulen et al, 1996). Mostly factor-VIII-related antibodies are used to stain endothelia, but this antibody may not highlight all intratumour microvessels, whereas CD31 antibodies are apparently more sensitive, in particular on paraffin-embedded sections (DeYoung et al, 1993; Weidner, 1995; Vermeulen et al, 1996). In order to obtain more objective data, we have, in addition to the classical blood vessel count, analysed the vessel density morphometrically using the same areas as selected for manual vessel counting. The data show a remarkable accordance of the values with only very few exceptions where vessel counting gave higher values than morphometric analysis. Comparing the results of both methods and in agreement with published data, we feel quite confident about the reliability of this information (Van Hoef et al, 1993; Barbareschi et al, 1995).

In contrast to skin SCCs, head and neck tumours tend to metastasize quite early and this may be associated with a different state of vascularization. In skin SCCs, the rate of metastasis is low, reaching only 4.5% in tumours exhibiting a thickness between 2 and 6 mm and approaching 15% in SCCs exceeding a thickness of 6 mm (Breuninger et al, 1990). In comparison, the usual TNM classification uses much larger dimensions (T1 < 2 cm; T2 > 2 cm) and is thus not appropriate for the microstaging of early skin carcinomas. In the absence of generally accepted criteria for evaluating early SCC stages we followed Breuniger's classification, which is considered more relevant than Broders' grading based on the degree of differentiation. Based on these criteria, we found an interesting correlation: the non-metastasizing carcinomas (<2 mm) did not show increased microvessel density. Only in those tumours exceeding 2 mm in thickness the vascular density was significantly increased. Notably, microvessel density was not influenced by the location of the tumour but just depended on its thickness and depth of penetration into the subepithelial stroma. This suggests that in skin SCCs increased vascular density might be a prognostic criterion and associated with tumour stages exhibiting increased metastatic probability.

The discrepancy between the experimental skin carcinogenesis data on transgenic mouse models (Hanahan and Folkman, 1996) and our data, may be partly explained by species differences and the discrepancy between virally- and UV-induced tumours. Importantly, experimentally induced mouse skin and genuine human tumours differ considerably in the latency periods of their development, i.e. by the different dynamics of tumour progression. On the other hand, skin epithelial tumours, as long as they remain surface tumours, may not require increased angiogenesis, as they are quite well nourished by diffusion similar to the situation of the parent tissue, the epidermis. Thus, increased angiogenesis would only be required with larger tumour masses, and consequently increased hypoxia within those areas (Detmar et al, 1997). On the other hand, angiogenesis and tumour vascularization may not only be required for nutrition and gas exchange but may actively participate in the invasion process by producing proteases or serving as trails for migrating tumour cells (Hamada et al, 1992; Rak et al, 1996; Skobe et al, 1997). Consequently, increased tumour vascularization would then not only be associated with enhanced invasion but may play an active part in tumour progression to the metastatic phenotype (Weidner, 1995; Ellis and Fidler, 1996). Thus, one may speculate that the late onset of increased tumour vascularization in skin SCCs may be associated with the low metastasizing capacity of these tumours.

### ACKNOWLEDGEMENTS

We thank Mrs Ursula Egner for technical assistance and advice, Dr Hans-Jörg Hacker for help in computer-assisted image analysis, Dr Hans-Jürgen Stark for help in immunohistologic- and Dr Rainer Spanbroek for help in in situ hybridization techniques as well as Mrs Martina Kegel for expert typing and Brigitte Nagel-Plagens for stylistic corrections. This work was supported by grants from the European Commission (BRPR-CT96-0227 and BIO4CT960464).

### REFERENCES

Barbareschi M, Weidner N, Gasparini G, Morelli L, Forti S, Eccher C, Fina P, Caffo O, Leonardi E, Mauri F, Bevilacqua P and Dalla Palma P (1995) Microvessel density quantification in breast carcinomas–assessment by light microscopy vs. a computer-aided image analysis system. *Appl Immunohistochem* **3**: 75–84

Barnhill RL, Fandrey K, Levy MA, Mihm MC and Hyman B (1992) Angiogenesis and tumour progression of melanoma–quantification of vascularity in melanocytic nevi and cutaneous malignant melanoma. *Lab Invest* **67**: 331–337

Barnhill RL and Levy M (1993) Regressing thin cutaneous malignant melanomas (1 mm) are associated with angiogenesis. *Am J Pathol* **143**: 99–104

Boukamp P, Peter W, Pascheberg U, Altmeier S, Fasching C, Stanbridge EJ and Fusenig NE (1995) Step-wise progression in human skin carcinogenesis in vitro involves mutational inactivation of p53, ras H oncogene activation and additional chromosome loss. *Oncogene* **11**: 961–969

Breuninger H, Black B and Rassner G (1990) Microstaging of squamous cell carcinomas. *Am J Clin Pathol* **94**: 624–627

© 2000 Cancer Research Campaign 

Broders AC (1932) Practical points on microscopic grading of carcinoma. *N Y State J Med* **32**: 667–671
Brown LF, Yeo KT, Berse B, Yeo TK, Senger DR, Dvorak HF and Van de Water L (1992) Expression of vascular permeability factor (vascular endothelial growth factor) by epidermal keratinocytes during wound healing. *J Exp M* **176**: 1375–1379
Callen JP (1991) Possible precursors to epidermal malignancies. In: *Cancer of the Skin*, Friedman RJ, Rigel DS, Kopf AW, Harris MN and Baker D (eds), pp. 27–34. WB Saunders: Philadelphia
Carrau RL, Barnes EL, Snyderman CH, Petruzelli G, Kachman K, Rueger R, D'Amico F and Johnson JT (1995) Tumor angiogenesis as a predictor of tumor aggressiveness and metastatic potential in squamous cell carcinoma of the head and neck. *Invasion Metastasis* **15**: 197–202
Coussens LM, Hanahan D and Arbeit JM (1996) Genetic predisposition and parameters of malignant progression in K14-HPV 16 transgenic mice. *Am J Pathol* **149**: 1899–1917
Detmar M, Brown LF, Claffey KP, Yeo KT, Kocher O, Jackman RW, Berse B and Dvorak HF (1994) Overexpression of vascular permeability factor/vascular endothelial growth factor and its receptors in psoriasis. *J Exp Med* **180**: 1141–1146
Detmar M, Kiang-Teck Y, Nagy JA, Van de Water L, Brown LF, Berse B, Elicker BM, Ledbetter S and Dvorak HF (1995) Keratinocyte-derived vascular permeability factor (vascular endothelial growth factor) is a potent mitogen for dermal microvascular endothelial cells. *J Invest Dermatol* **105**: 44–50
Detmar M, Brown LF, Berse B, Jackman RW, Elicker BM, Dvorak HF and Claffey KP (1997) Hypoxia regulates the expression of vascular permeability factor/vascular endothelial growth factor (VPF/VEGF) and its receptors in human skin. *J Invest Dermatol* **108**: 263–268
DeYoung BR, Wick MR, Fitzgibbon JF, Sirgi KE and Swanson PE (1993) CD31: an immunospecific marker for endothelial differentiation in human neoplasms. *Appl Immunohistochem* **1:** 97–100
Ellis LM and Fidler IJ (1996) Angiogenesis and metastasis. *Eur J Cancer* **32A**: 2451–2460
Folkman J (1995) Angiogenesis in cancer, vascular, rheumatoid and other disease. *Nat Med* **1**: 27–31
Fusenig NE and Boukamp P (1998) Multiple stages and genetic alterations in immortalization, malignant transformation, and tumour progression of human skin keratinocytes. *Mol Carcinogen* **23**: 144–158
Gasparini G, Weidner N, Maluta S, Pozza F, Boracchi P, Mezzetti M, Testolin A and Bevilacqua P (1993) Intratumoral microvessel density and p53 protein: correlation with metastasis in head and neck squamous cell carcinoma. *Int J Cancer* **55**: 739–744
Goepfert H, Dichtel W, Medina JE, Landberg RD and Luna MD (1984) Perineural invasion of SCC of the head and neck. *Am J Surg* **148**: 542–547
Graham CH, Rivers J, Kerbel RS, Stankiewicz KS and White WL (1994) Extent of vascularization as an independent prognostic indicator in thin (0.76 mm) malignant melanomas. *Am J Pathol* **145**: 510–514
Guidi AJ, Fischer L, Harris JR and Schnitt SJ (1994) Microvessel density and distribution in ductal carcinoma in situ of the breast. *J Natl Cancer Inst* **86:** 614–619
Hamada J, Cavanaugh PG, Lotan O and Nicolson GL (1992) Separable growth and migration factors for large-cell lymphoma cells secreted by microvascular endothelial cells derived from target organs for metastasis. *Br J Cancer* **66**: 349–354
Hanahan D and Folkman J (1996) Patterns and emerging mechanisms of the angiogenic switch during tumorigenesis. *Cell* **86**: 353–364
Hsu SM and Soban E (1982) Color modification of diaminobenzidine (DAB) precipitation by metallic ions and its application for double immunohistochemistry. *J Histochem Cytochem* **30**: 1079–1082
Keck PJ, Hauser SD, Krivi G, Sanzo K, Waren T, Feder J and Connolly DT (1989) Vascular permeability factor, an endothelial cell mitogen related to PDGF. *Science* **246**: 1309–1312
Kerschmann RL, McCalmont TH and LeBoit PE (1994) p53 oncoprotein expression and proliferation index in keratoacanthoma and squamous cell carcinoma. *Arch Dermatol* **130**: 181–186
Marcoval J, Moreno A, Graells J, Vidal A, Escriba JM, Garcia-Ramirez M and Fabra A (1997) Angiogenesis and malignant melanoma. Angiogenesis is related to the development of vertical (tumorigenic) growth phase. *J Cutan Path* **24**: 212–218
Marks R, Rennie G and Selwood TS (1988) Malignant transformation of solar keratoses to squamous cell carcinoma. *Lancet* **1**: 795–797
Moorman AFM, De-Boer PAJ, Vermeulen JLM and Lamers WH (1992) Practical aspects of radio-isotopic in situ hybridization on RNA. *Histochem J* **25**: 251–266
Pyke C, Kristensen P, Ralfkiaer E, Eriksen J and Dano K (1991) The plasminogen activation system in human colon cancer: messenger RNA for the inhibitor PAI-1 is located in endothelial cells in the tumour stroma. *Cancer Res* **51**: 4067–4071
Rak J, Filmus J and Kerbel RS (1996) Reciprocal paracrine interactions between tumour cells and endothelial cells: the 'angiogenesis progression' hypothesis. *Eur J Cancer* **32A**: 2438–2450
Schlenger K, Höckel M, Mitze M, Schäffer J, Weikel W, Knapstein PG and Lambert A (1995) Tumor vascularity – a novel prognostic factor in advanced cervical carcinoma. *Gynecol Oncol* **59**: 57–66
Skobe M, Rockwell P, Goldstein N, Vosseler S and Fusenig NE (1997) Halting angiogenesis suppresses carcinoma cell invasion. *Nature Med* **3**: 1222–1227
Smith-McCune K and Weidner N (1994) Demonstration and characterization of the angiogenic properties of cervical dysplasia. *Cancer Res* **54**: 800–804
Smith-McCune K, Zhu Y-H, Hanahan D and Arbeit J (1997) Cross-species comparison of angiogenesis during the premalignant stages of squamous carcinogenesis in the human cervix and K14-HPV16 transgenic mice. *Cancer Res* **57**: 1294–1300
Srivastava A, Laidler P, Davies RP, Horgan K and Hughes LE (1988) The prognostic significance of tumour vascularity in intermediate-thickness (0.76–4.0 mm thick) skin melanoma. *Am J Pathol* **133**:419–423
Staibano S, Boscaino A, Salvatore G, Orabona P, Palombini L and De Rosa G (1996) The prognostic significance of tumour angiogenesis in nonaggressive and aggressive basal cell carcinoma of the human skin. *Hum Pathol* **27**: 695–700
Tahan SR and Stein AL (1995) Angiogenesis in invasive squamous cell carcinoma of the lip: tumor vascularity is not an indicator of metastatic risk. *J Cutan Path* **22**: 236–240
Urbach F and Graham JH (1962) Anatomy of human skin tumour capillaries. *Nature* **194**: 652–654
Van Hoef MEHM, Knox WF, Dhesi SS, Howell A and Schor AM (1993) Assessment of tumour vascularity as a prognostic factor in lymph node negative invasive breast cancer. *Eur J Cancer* **29A**: 1141–1145
Vermeulen PB, Gasparini G, Fox SB, Toi M, Martin L, McCulloch P, Pezella F, Viale G, Weidner N, Harris AL and Dirix LY (1996) Quantification of angiogenesis in solid human tumours: an international consensus on the methodology and criteria of evaluation. *Eur J Cancer* **32A**: 2474–2484
Weedon D (1997) Tumors of the epidermis. In: *Skin Pathology*, Weedon D (ed) pp. 642–671. Churchill Livingstone, Edinburgh
Weidner N (1995) Intratumor microvessel density as a prognostic factor in cancer. *Am J Pathol* **147**: 9–19
Weidner N, Semple JP, Welch WR and Folkman J (1991) Tumour angiogenesis and metastasis – correlation in invasive breast carcinoma. *N Engl J Med* **324**: 1–8
Weninger W, Uthman A, Pammer J, Pichler A, Ballaun C, Lang IM, Plettenberg A, Bankl HC, Stürzl M and Tschachler E (1996) Vascular endothelial growth factor production in normal epidermis and in benign and malignant epithelial skin tumours. *Lab Invest* **75**: 647–657
Weninger W, Rendl M, Pammer J, Grin W, Petzelbauer P and Tschachler E (1997) Differences in tumor microvessel density between squamous cell carcinomas and basal cell carcinomas may relate to their different biologic behavior. *J Cutan Pathol* **24**: 364–369
Zätterström UK, Brun E, Willen R, Kjellen E and Wennerberg J (1995) Tumor angiogenesis and prognosis in squamous cell carcinoma of the head and neck. *Head Neck* **17**: 312–318
Ziegler A, Jonason AS, Leffell DJ, Simon JA, Sharma HW, Kimmelman J, Remington L, Jacks T and Brash DE (1994) Sunburn and p53 in the onset of skin cancer. *Nature* **372**: 773–776

 *© 2000 Cancer Research Campaign*
